# Angle Accuracy of Intramedullary Bone Resection Guides for Total Knee Arthroplasty

**DOI:** 10.7759/cureus.68769

**Published:** 2024-09-06

**Authors:** Matthew C Kane, Harold E Cates, Iou-Ren Chang

**Affiliations:** 1 Surgery, East Tennessee State University Quillen College of Medicine, Johnson City, USA; 2 Orthopedic Surgery, Tennessee Orthopedic Foundation for Education and Research, Knoxville, USA; 3 Orthopedic Surgery, OhioHealth Doctors Hospital, Columbus, USA

**Keywords:** total knee arthroplasty technique, robotic total knee arthroplasty, angle accuracy, total knee arthroplasty (tka), intramedullary guide

## Abstract

The importance of proper prosthetic placement has been confirmed in numerous studies. The objective of this study was to compare the planned resection angles to the verified intraoperative angles of femoral and tibial varus/valgus, tibial slope, and femoral flexion for each total knee performed using intramedullary (IM) cut guides for both distal femur and proximal tibia cuts. A total of 1,000 total knee arthroplasties (TKAs) were evaluated for this study. Intraoperative cut-check technology was used to show real-time validation of these resection angles. Assuming an acceptable range of within 2° of the planned cuts, results show the femoral varus/valgus angles were 75% accurate, the femoral flexion angles were 50.8% accurate, the tibial cuts were 95.2% accurate in the coronal plane, and the tibial slope was the least accurate with only 50.3% within the acceptable range. This showed that IM guides are reasonably accurate in producing desired angles in the coronal plane but less accurate in the sagittal plane, with a greater number of outliers in femoral flexion and posterior slope. Surgeons need to be aware of potential cutting errors when using IM guides as they affect the overall alignment of the implant, and real-time verification technology is available to verify the accuracy of the cuts.

## Introduction

The commercial availability of total knee arthroplasty (TKA) surgery began in the early 1970s. Since that time, orthopedic surgeons and researchers have worked diligently to improve the accuracy of bone cuts and implant placement in attempts to minimize component failure and produce greater clinical outcomes. Early studies in biomechanics recognized prosthetic obliquity and calculated the shear load developed by angular tibial prosthetic placement [1]. The importance of proper prosthetic placement has been confirmed in numerous studies, including a 2004 study that identified component malposition as a cause of loosening and osteolysis [2]. Variance in either the coronal or sagittal planes can lead to early failure. Increasing the tibial posterior slope can produce anterior micro-motions of the tibia component [3]. As little as 3° variance in the sagittal plane of either the distal femur or proximal tibia cuts can significantly change the total load on the medial and lateral compartments of the tibia [4]. One study has been published claiming that proper alignment has little effect on 15-year survival rates for cemented total knee replacements. This study only considered failure rates of knee replacements with a postoperative mechanical axis between 0° and 3° versus failure rates of outliers [5]. Notably, the majority of studies, including a commonly cited Ritter article, agree that malaligned knees have a higher revision rate than properly aligned knees [6]. Major implications resulting from minor variations exemplify the importance of surgeon accuracy when planning and performing knee replacement surgery.

Today, intramedullary (IM) cut guides remain the most commonly used system for primary cuts to the distal femur, while many surgeons use extramedullary guides for proximal tibia cuts. We previously compared the accuracy of cuts using IM and extramedullary tibial cutting guides. The findings showed greater accuracy in both planes using IM versus extramedullary guides [7]. Due to prior published results, the author primarily prefers to continue to use IM guides for the femur and tibia. There remains a small yet significant range of cuts outside of an acceptable variance that may require a recut to achieve a satisfactory component position. The development of new technology can aid surgeons in creating more accurate cuts, recognizing unplanned variances, and making necessary changes intraoperatively. Current intraoperative cut check technology, such as the Stryker Navigation System, allows real-time validation of these resection angles.

The objective of this study is to document the range of variability for IM cutting guides and to make surgeons aware of the potential use of real-time cut checks to improve their prosthetic positioning. Data were collected from a single surgeon performing TKAs from 2008 to 2020. The selected data were extracted from op notes for patients on whom the initial cut for both the distal femur and proximal tibia was done using IM cut guides and which had recorded planned versus actual cut angles as determined intraoperatively using the Stryker Navigation System. This is a 1,000-knee study determining cut variability in four categories: (1) femoral flexion/extension, (2) femoral varus/valgus, (3) tibial posterior slope, and (4) tibial varus/valgus.

## Materials and methods

A single-surgeon study was performed using data collected from primary TKA procedures completed between 2008 and 2020. During this period, the surgeon used the measured resection technique to plan and perform distal femoral and proximal tibial cuts. A variety of IM cut guide systems were utilized, including Zimmer Persona, Zimmer Nexgen, Biomet Vanguard, Smith & Nephew Journey II, Smith & Nephew Legion, and Stryker Triathlon. The planned cut angles were recorded preoperatively, and the settings were checked in the surgical field. Following the primary resection, the Stryker (ASM) Navigation System was used to determine the actual cut angles, and these values were recorded.

Computer navigation is a safe and accurate way to verify cut angles intraoperatively [8]. If the variance was outside of the acceptable range, a recut was initiated when indicated by the overall lower extremity alignment. This study is only concerned with the primary resection angles. Further study would need to be done to determine the accuracy of secondary resections. Patient identifying information was removed from the op notes so that research could be done strictly based on cut angle accuracy.

Four thousand five hundred cases were reviewed for data collection. Data were only considered for this study if IM guides were used for both the primary distal femoral resection and the primary proximal tibia resection. Patients on whom extramedullary guides, custom guides, or robotic computer-based cut guides were used were excluded from this study, as well as any patients who did not have complete data for the four measurements being evaluated. The remainder provided data for the 1,000-knee study.

Specific equations used to calculate variance for each cut angle can be found in Appendix A. An overview of each cut angle variance is described below.

The planned angle for femoral flexion was 0°. However, based on the author’s previous experience, variance of up to 2° in either direction was considered acceptable. In some cases, greater variances in the direction of added flexion would not require a recut, but for the purposes of this study, any variance greater than 2° was considered an outlier.

Calculating the variance in femoral varus/valgus was slightly more complex than other measurements since the IM guides are based on anatomical alignment and ASM cut checks are based on mechanical alignment. The assumed standard for anatomical alignment is 7° valgus. Therefore, an IM femoral guide set at 5° valgus is equivalent to 2° varus relative to the mechanical axis. After making each resection, the Stryker Navigation System was again used intraoperatively to check the actual resection angle. A variance of up to 2° was determined to be within an acceptable range. If unacceptable angles were found, recuts were made, and the cuts were checked again. These variances had to be hand calculated since the desired value was a directional shift. For example, if the planned resection was 2° varus and the actual resection angle was 5° varus, the variance would be 3° in the varus direction. Similarly, if the planned resection was 2° varus and the actual resection angle was 1° valgus, the variance would be 3° in the valgus direction. If the planned and actual resection angles were the same, it was recorded as 0° variance.

For tibial posterior slope variance, actual values for the posterior slope were subtracted from a preoperative plan of either 0°, 3°, or 5° (posterior). A variance in slope up to 2° in either direction was considered acceptable.

The varus/valgus planned tibial cut was 0°. Therefore, actual cut angles were recorded as either varus or valgus as they shifted from 0°. Acceptable variances were within 2° varus or valgus. Variances in the varus direction were given a negative value, while variances in the valgus direction were kept positive for the purpose of analyzing the directional shifts.

Each set of 1,000 data points was used to create a column graph containing a bell curve representing the variance of the actual cut angle from the planned cut angle. The bar at point 0 indicates no variance from the planned cut angle. There are also two vertical lines included in each graph representing the maximum acceptable variance for each resection angle. Any variance greater than 2° in either direction was considered an outlier.

## Results

**Figure 1:**
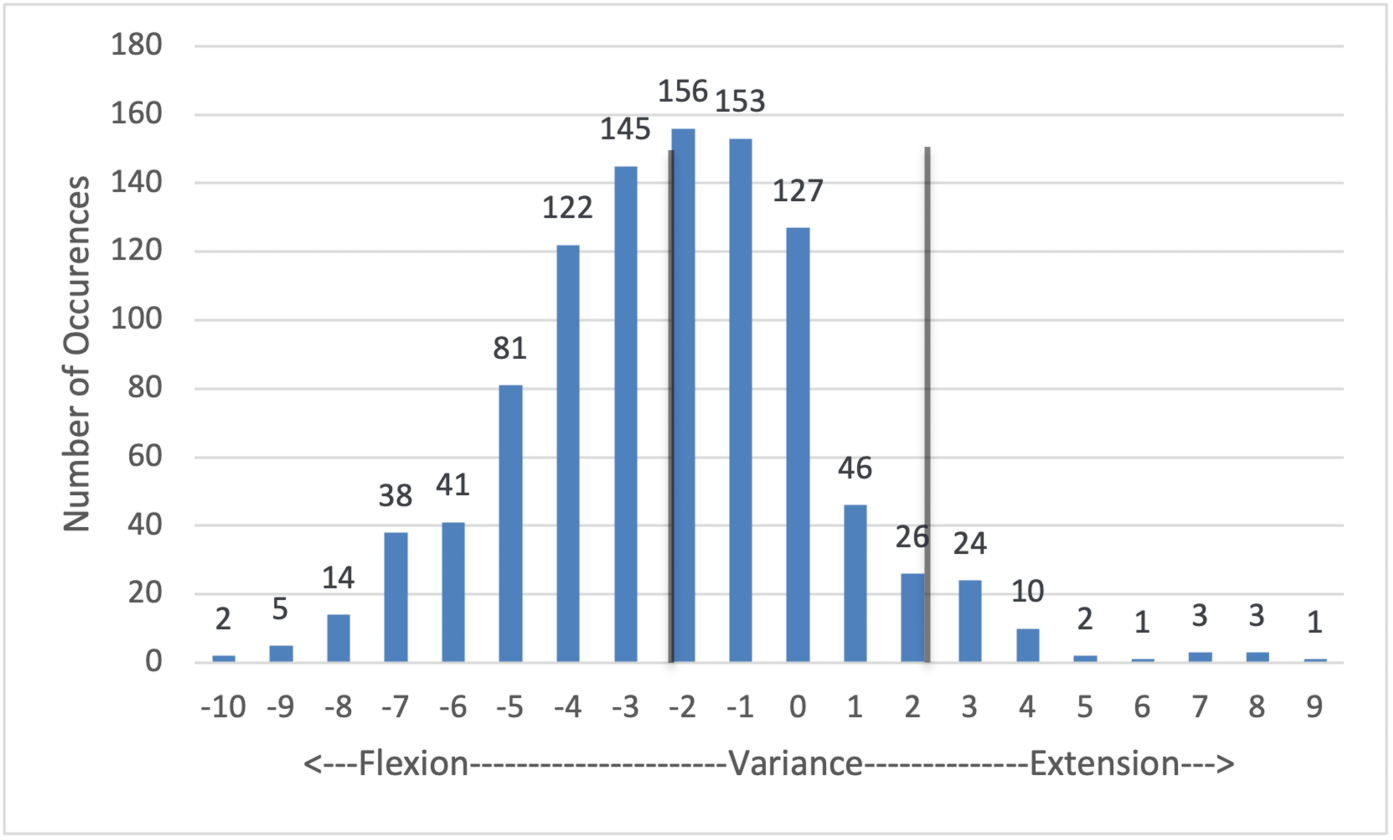
Femoral cut: flexion/extension variance Values that fall between the vertical gray lines are within 2° of the planned cut angle and were deemed within the acceptable range of variance. Values outside of this range were considered outliers and were subject to recut at the surgeon's discretion.

Femoral flexion (Figure 1): There is a tendency here to vary in the direction of added flexion; 508/1,000 (50.8%) data points fall between 2° of flexion and 2° or extension, again with the majority, 309/508 in the direction of increased flexion. There is also a significant number, 441/1,000, that falls between 3° and 8° of added flexion. Outliers reached a 10° variance in this plane, and 492/1,000 (49.2%) fell outside of the acceptable range of variability, requiring consideration for secondary cuts.

**Figure 2:**
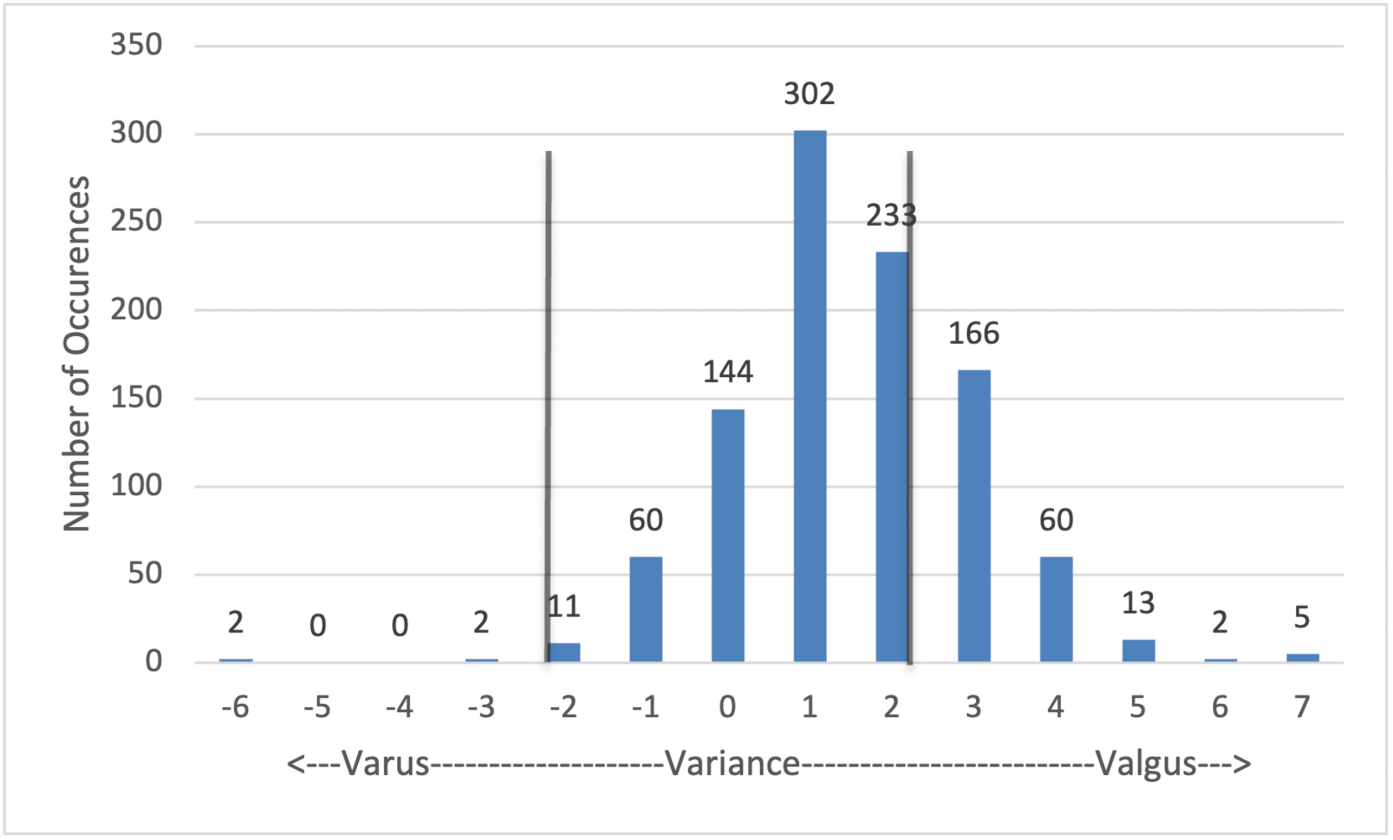
Femoral cut: varus valgus variance Values that fall between the vertical gray lines are within 2° of the planned cut angle and were deemed within the acceptable range of variance. Values outside of this range were considered outliers and were subject to recut at the surgeon's discretion.

Femoral varus/valgus (Figure 2): 750/1,000 (75%) cuts were within the allowance of 2° variance either in the varus or valgus directions. The variance here was shifted in the valgus direction, with 535/1,000 cuts varying between 1° and 2° valgus and 226/1,000 cuts varying between 3° and 4° valgus. Outliers here only reached 7° variance; however, only 250/1,000 (25%) of measurements in this plane fell outside of the acceptable range of variability and would require consideration for recuts.

**Figure 3:**
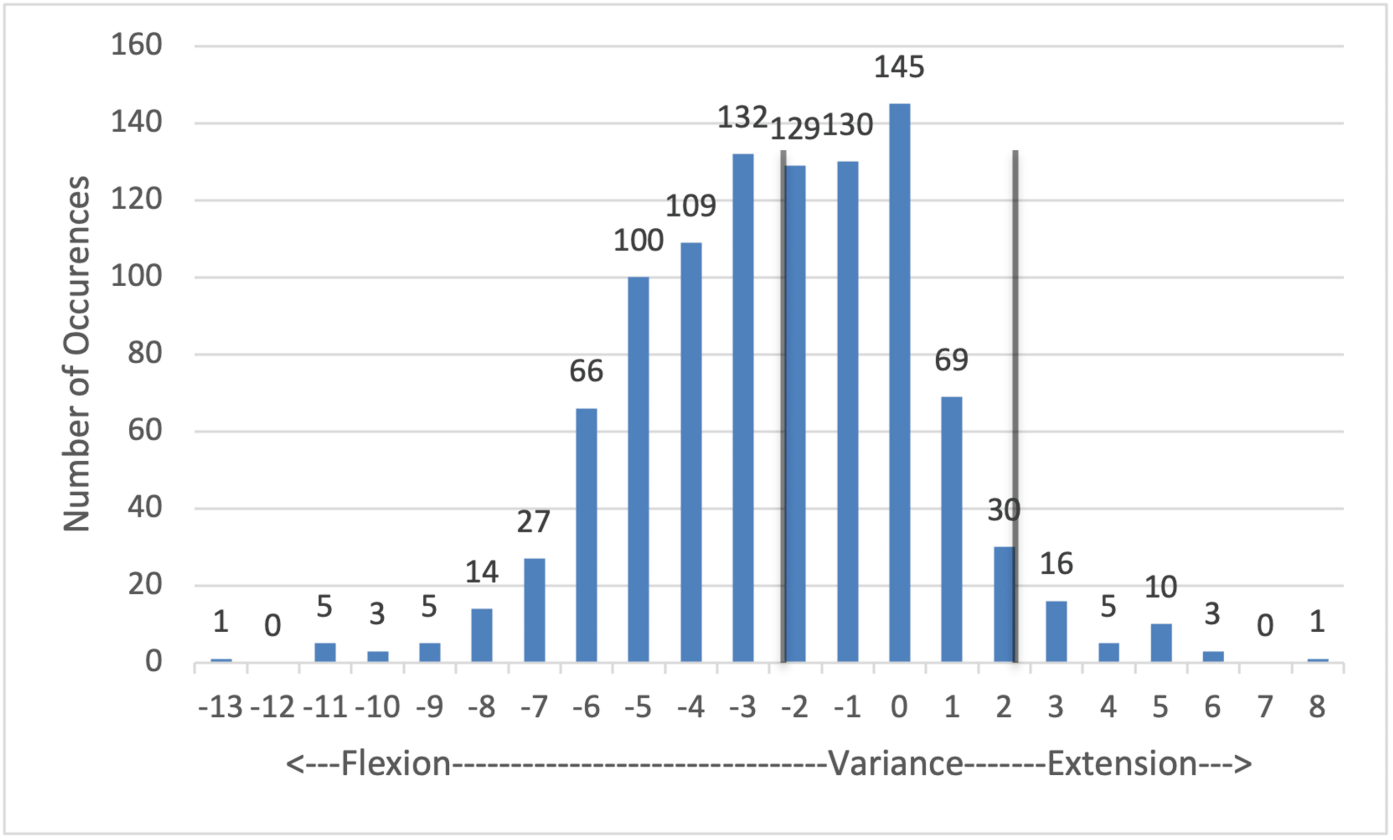
Tibial cut: posterior slope variance Values that fall between the vertical gray lines are within 2° of the planned cut angle and were deemed within the acceptable range of variance. Values outside of this range were considered outliers and were subject to recut at the surgeon's discretion.

Post-slope (Figure 3): There again is a tendency to vary in the direction of increased flexion. 503/1,000 (50.3%) points fall between 2° of added flexion and 2° of added extension, but the majority, 259/503, are on the side of increased flexion. There is also a significant number, 434/1,000, that falls between 3° and 7° of added flexion. Outliers reached as far as 13° variance. In total, 497/1,000 (49.7%) of measurements in this plane fell outside of the acceptable range of variability, requiring consideration for secondary cuts.

**Figure 4:**
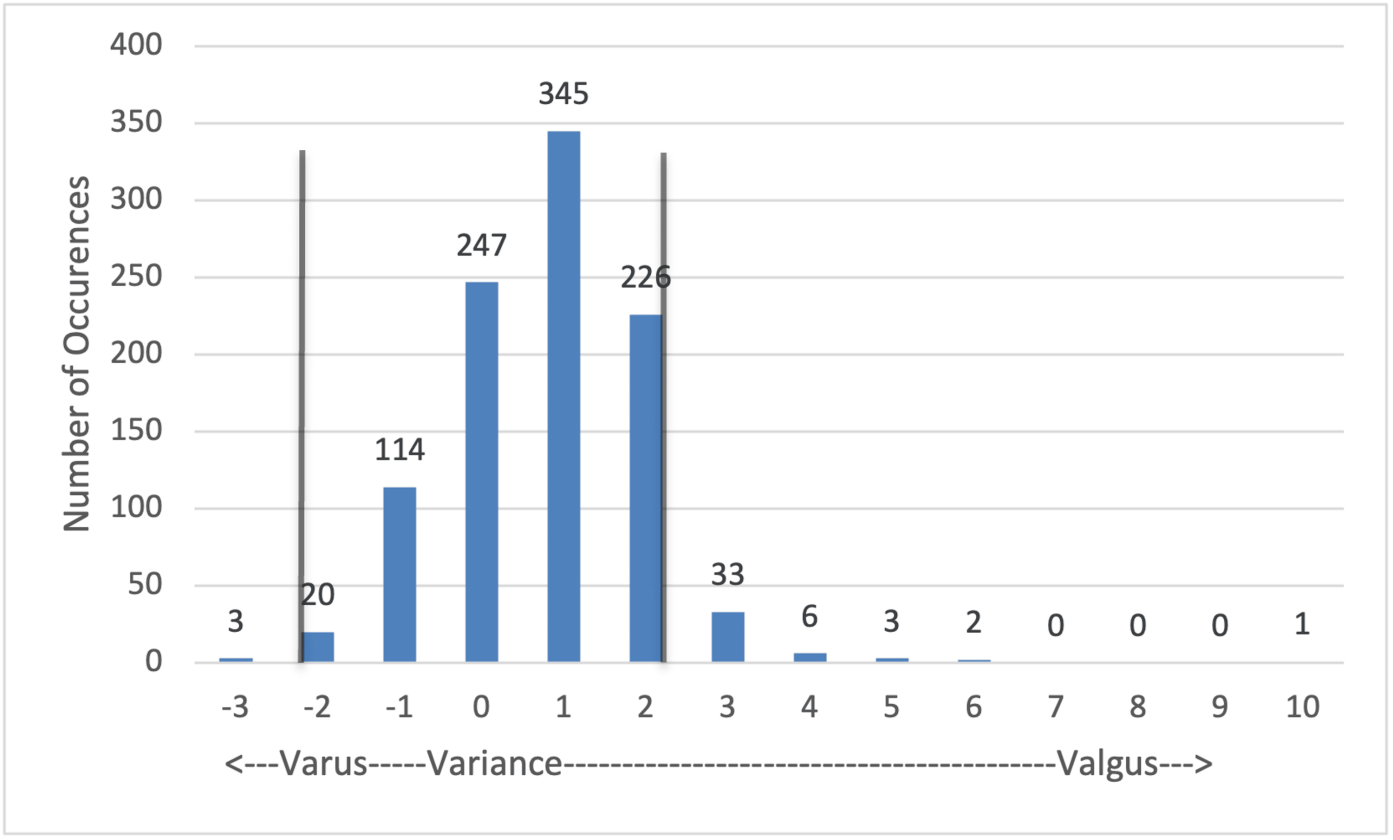
Tibial cut: varus valgus variance Values that fall between the vertical gray lines are within 2° of the planned cut angle and were deemed within the acceptable range of variance. Values outside of this range were considered outliers and were subject to recut at the surgeon's discretion.

Tibial varus/valgus (Figure 4): 952/1,000 (95.2%) cuts were within the allowance of 2° variance either in the varus or valgus directions. The greatest occurrence was a 1° variance in the varus direction, 345/1,000 cuts. Outliers reached as far as 10° variance, while only 48/1,000 (4.8%) of measurements in this plane fell outside of the acceptable range of variability.

## Discussion

There are many factors that contribute to resection angle variability. These may include placement of the IM rod, femoral bow, starting point of the IM rod, toggle of the saw blade, pin placement, individual anatomic variation, tibial anatomy, and previous deformity. Each of these factors must be considered when performing IM-guided resections and when analyzing variances for either femoral or tibial cuts.

Variances to femoral resections have a wider range of acceptability and require fewer recuts than tibial resections. Figure 1 shows a true bell-shaped curve peaking at 2° of added flexion, but with the majority of cuts (50.8%) within the acceptable range of variability; 49.2% of cuts were outside of the range of ±2° of flexion or extension. Notably, the vast majority of cuts added flexion to the planned cut. While a wide range is noted, alignments varying in this plane are more acceptable, so additional flexion in the distal femoral resection is rarely a cause for secondary resection [9].

Varus/valgus variance in the distal femoral resection created a sharp spike, as shown in Figure 2, indicating a higher degree of accuracy in the coronal plane. The peak variance was 1° valgus from the planned cut, and only 25% of cuts fell outside of acceptable parameters of 2° in either direction. Variance in the valgus direction would be less problematic according to previous studies, which state that it is better to align the knee either neutral or slightly valgus for the greatest TKA survival rates [9]. Specific to the femur, variances can be attributed to the entry point of the IM guide, femoral bow, pin placement, and anatomic versus mechanical axis angles.

The proximal tibia resections showed the greatest variance in the posterior slope measurement. Figure 3 shows that 49.7% of resections of the tibia were outside of the acceptable parameters of 2° of added slope posteriorly or anteriorly. It is more vital that the tibia slope be closer to the planned angle since a steeper tibial slope can create micromovements of the implants on the insert. This can also lead to adverse kinematic patterns as well as positions outside ideal product specifications for tibial poly positioning, which can lead to abnormal stress on the poly. Specific to the tibial slope, variations to the tibial anatomy, entry point of the IM rod, and pin placement contribute to variance in the resection angle.

As was observed with femoral cuts, variance in the coronal plane of the tibia was significantly lower than in the sagittal plane. Figure 4 shows that the data points peak again at 1° of valgus, and only 4.8% of cuts vary outside of 2° in either direction. Contributors to these variances also include IM rod placement, tibial anatomy, and pin placement.

This study shows that there is a significant variation in bone resection angles when utilizing IM cutting guides. If unchecked, there may be unexpected outcomes. The results confirm previous accuracy studies that showed similar variance in cuts using IM guides [7,10-11]. These studies show that variances have changed very little in the last 30 years. Variability was higher in the sagittal plane than the coronal plane, consistent with these studies and others, including a 2001 study that credited 10-40% of variance to guide movement [12]. A previous study performed by the author showed similar variability for IM-guided femoral and tibial cuts, using postoperative imaging to compare planned and actual angles [7]. The advantage of this study was the use of real-time cut checks with the Stryker Navigation System, which provides more accurate cut angle measurements and allows for changes to be made intraoperatively.

Other studies confirm guide movement as a cause for error, as well as the toggle of the saw blade within the slot [13]. Guide movement could originate from pin placement or could result from torquing of the saw while making the cut. Regardless of origin, these movements lead to inaccuracies in bone resection that could result in malpositioned components. The vast majority of studies agree that malpositioned components are problematic, causing loosening, osteolysis, and, in some cases, early component failure [1,2,6,11]. One study claims that malpositioned components have no effect on outcomes when considering only the coronal alignment of the femur [5]. Using intraoperative cut checks can help determine if a variance may have detrimental results. It should also be considered that all of these variables have sum ranges. For example, if the femoral cut varies by 2° valgus and the tibial cut varies by 2° valgus, the sum would be 4° of overall valgus alignment. For this reason, checking all angles during the intraoperative decision-making process can be advantageous to achieving acceptable overall knee alignment.

Other factors also contribute to component malposition. Patterns of femoral component malposition have been shown to occur even with precise instrumentations [14]. Reliance on fixed resection angles for femoral cuts has proven problematic for establishing proper femoral mechanical alignment [15]. These errors were not addressed in this study, only inaccuracies contributed by variance in resection angles while using IM guides. This study does show that there is a limit to the dependence that should be placed on IM cut guides. Understanding these limits, real-time cut checks allow surgeons to be more precise and make well-informed decisions intraoperatively [16].

The strengths of this study are that a single surgeon performed every resection, and a single validation tool (Stryker Navigation System) was used throughout the entire process, minimizing variation in technique and providing consistent measurements of resection angles. A weakness, however, is that it is not specific to one IM guide system. While this study shows the variance of resection angles using IM guides in general, prior analysis showed no significant difference between the different company’s IM guides.

One method of dealing with possible variances is to use real-time cut checks to validate resections. Other methods may include custom guides, patient-specific instrumentation, and the use of robotic assistance. These methods are becoming increasingly common, and some have reported a higher rate of accuracy when using these techniques [10]. Understanding that no system is perfect, verifying angles intraoperatively allows the surgeon to make well-informed decisions in real time.

## Conclusions

Understanding the limitations of instrumentation can help surgeons more accurately place TKA implants. IM guides continue to be used despite shortcomings. This study found that variations outside of an acceptable range occurred in 49.2% of distal femoral cuts in reference to adding flexion or extension, 25% of distal femoral cuts in reference to varus/valgus orientation, 49.7% of proximal tibia cuts in reference to posterior slope, and 4.8% of proximal tibia cuts in reference to varus/valgus orientation. It can benefit surgeons to use real-time cut checks to verify their resection angles and determine when and where adjustments need to be made.

## Appendices

Equations

*Femoral Flexion:* Variance = Planned (0°) - Actual

     (-) = flexion, (+) = extension

*Femoral Varus/Valgus:* Variance = directional shift from Planned to Actual, accounting for mechanical axis:

     Example 1: Planned = 7° - (5° valgus) = 2° varus, Actual = 1° valgus,

                   Then: 2° varus --> 1° valgus = 3° valgus

     Example 2: Planned = 7° - (5° valgus) = 2° varus, Actual = 4° varus,

                    Then: 2° varus --> 4° varus = 2° varus

*Tibial Posterior Slope:* Variance = Planned (0°, 3°, or 5°) - Actual

     (-) = flexion, (+) = extension

*Tibial Varus/Valgus:* Variance = directional shift from 0°

     (-) = varus, (+) = valgus
